# Doxycycline-induced ulceration mimicking esophageal cancer

**DOI:** 10.1186/1757-1626-1-144

**Published:** 2008-09-08

**Authors:** Veysel Tahan, Hakan Sayrak, Nevzat Bayar, Burak Erer, Gulgun Tahan, Faysal Dane

**Affiliations:** 1 Department of Gastroenterology, Pasabahce State Hospital, Istanbul, Turkey; 2 Department of Pathology, Pasabahce State Hospital, Istanbul, Turkey; 3 Department of Internal Medicine, Pasabahce State Hospital, Istanbul, Turkey; 4 Department of Internal Medicine Umraniye State Hospital, Istanbul, Turkey; 5 Surgery Unit, Institute of Gastroenterology, Marmara University, Istanbul, Turkey; 6 Department of Oncology, Marmara University School of Medicine, Istanbul, Turkey

## Abstract

**Introduction:**

Doxycycline-induced esophageal ulcer patients are mostly young persons with no history of esophageal dysfunction. Heartburn, midsternal pain and dysphagia are the most common symptoms. It has generally a benign course. The present case is the first report of doxycycline-induced extensive ulcerations, mimicking esophageal cancer in two esophageal segments alongside, in the literature.

**Case presentation:**

This report describes a 16-year-old Caucasian girl who, while taking doxycycline capsules100 mg twice a day for acne vulgaris for 3 months, developed these symptoms. An upper endoscopy revealed multiple circumferential deep ulcerations surrounding fragile, irregular, hyperemic and hypertrophic mucosa at the level of the mid-esophagus and concomitantly in the lower esophageal sphincter. The lesions were biopsied to exclude esophageal carcinoma because of the suspicious appearance in the endoscopic examination. The histopathological examination, haematoxylin and eosin stained sections showed ulceration with a mixed inflammatory infiltrate. Doxycycline was discontinued and she was given sucralfate 1 g qid and omeprazole 20 mg bid orally. All symptoms of the patient were resolved on the third day of the treatment. After 4 weeks of the therapy, an upper endoscopic control examination demonstrated normal findings.

**Conclusion:**

The present case has been an uncommon presentation of doxycycline-induced extensive ulcerations, mimicking esophageal cancer in two esophageal segments, concomitantly. Even the lesions were biopsied to exclude esophageal carcinoma. A modification on the behavior of taking drugs can prevent these unpleasant complications.

## Introduction

Doxycycline-induced esophageal lesions had been examined mostly as mild esophagitis and sometimes as ulceration with a generally benign course. Common reason of this complication has been taking medications just before bedtime, and with a small amount of water [1,2]. In general the certain drugs tend to cause the damage in a singular segment, as doxycycline in mid-esophageal segment [3]. Here we present a severe, unusual endoscopic appearance of a case of doxycycline-induced esophageal ulcers and tissue fragility mimicking esophageal cancer in the lower and mid-esophageal segments, simultaneously.

## Case report

**Figure 1:**
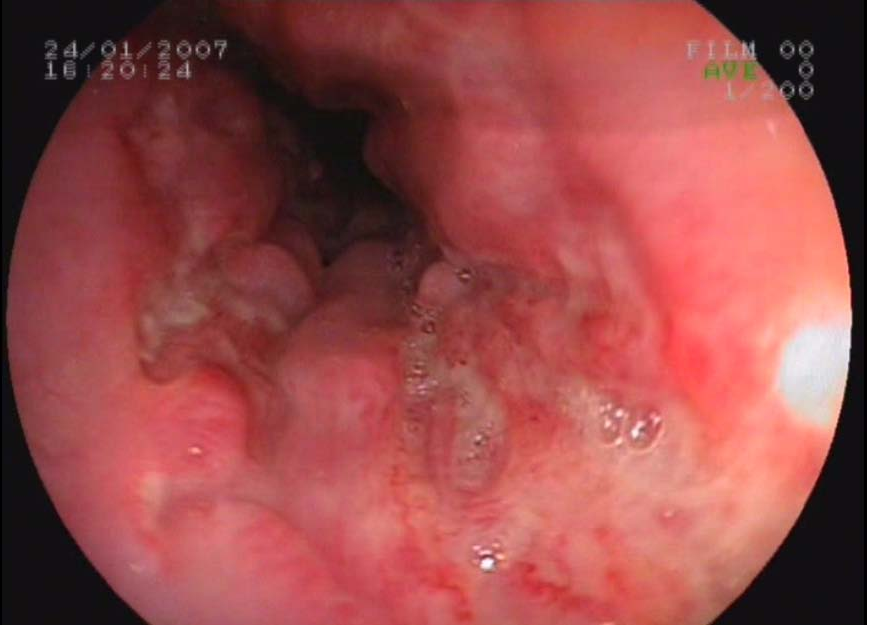
**Endoscopic appearance of the ulcers mimicking cancer at the level of the mid-esophagus**. An upper endoscopic examination revealed multiple circumferential deep ulcerations surrounding fragile, irregular, hyperemic and hypertrophic mucosa at the level of the mid-esophagus.

**Figure 2:**
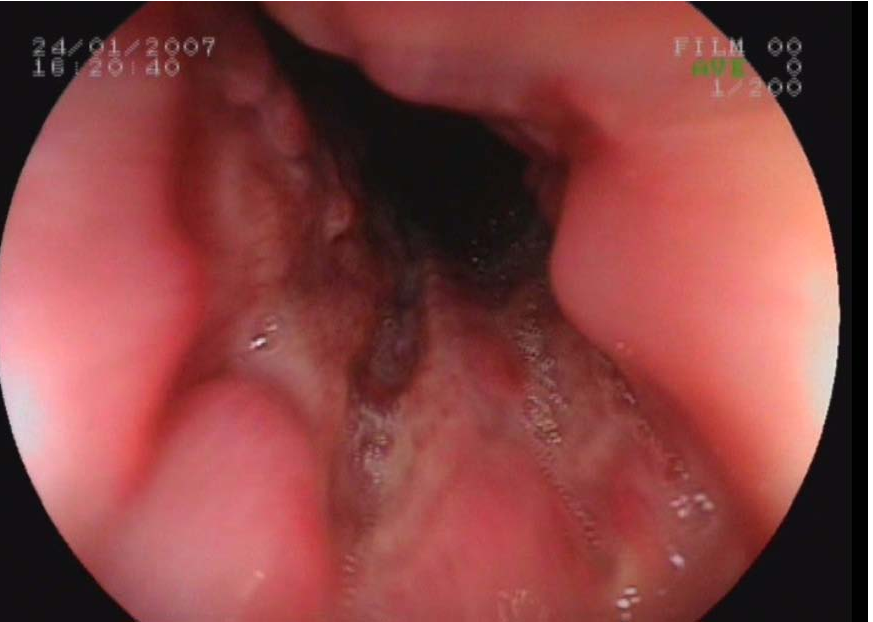
**Endoscopic appearance of the ulcers mimicking cancer in the lower esophageal sphincter**. Same endoscopic findings of the mid-esophagus were observed in the lower esophageal sphincter.

**Figure 3:**
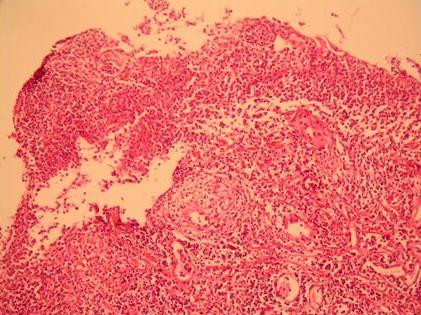
**Histopathological section of the ulcer tissue**. Histopathological section of the ulcer tissue showed ulceration with a mixed inflammatory infiltrate (HE, × 100).

A 16-year-old Caucasian girl was admitted to our outpatient clinic with complains of heartburn, midsternal pain and dysphagia for 4 weeks. She had no fever, cough or any additional complain. Her cardiac and chest auscultation and throat examination were normal. Her whole blood count and erythrocyte sedimentation rate were also normal. Her electrocardiography and chest x-ray revealed no pathological findings. An upper endoscopy revealed multiple circumferential deep ulcerations surrounding fragile, irregular, hyperemic and hypertrophic mucosa at the level of the mid-esophagus (Figure 1) and lower esophageal sphincter (Figure 2). Because of the suspicious endoscopic appearance, the lesions were biopsied to exclude esophageal carcinoma. In her detailed history, she was taking doxycycline capsules 100 mg twice a day for acne vulgaris for 3 months. She was swallowing only the capsule in the recumbent position at midnight. In the histopathological examination, haematoxylin and eosin stained sections showed ulceration with a mixed inflammatory infiltrate (Figure 3). Doxycycline was discontinued and she was given sucralfate 1 g qid and omeprazole 20 mg bid orally to control of gastric acid reflux until her symptoms resolve. All symptoms of the patient were dramatically resolved on the third day of the treatment. A control endoscopy demonstrated normal findings, after 4 weeks of the therapy.

## Discussion

Drug-induced ulcer is thought not to be so common; however it is not an unusual clinical condition especially in patients taking certain drugs. Doxycycline-induced esophageal ulcer patients are mostly young persons with no history of esophageal dysfunction. The main reason for an increased transit time is to take the medication with a small amount of water immediately before sleeping, as the present case did [1,2]. Drug-induced esophagitis is more frequent with capsule than with tablet, because of its easier adhesion to the esophageal surface [4]. As an acidic drug, doxycycline accumulation in the epithelial cells can also cause a focal contact esophagitis. With its subsequent local cytochemical effects, doxycycline can cause ulceration and friability of the adjacent esophageal mucosa. In addition, doxycycline can also inhibit protein synthesis functions in the esophagus [5,6].

Nevertheless history and upper endoscopy can confirm the diagnosis in almost all cases. In suspected conditions, exclusion of esophageal carcinoma by histology can be necessary [2], as it was the case in our patient.

Gencosmanoglu et al. revealed that tetracycline tends to cause distal esophagitis and doxycycline is more frequently associated with mid-esophageal ulceration [3]. However, in the present case, we identified doxycycline-induced ulcers in both distal and mid-esophageal localizations. Drug-induced esophagitis should be suspected in all patients presenting with chest pain and dysphagia and is a preventable cause of morbidity that consists of giving simple advice of how and when to take medication. The prevention could be achieved by swallowing the drug with at least 100 ml of water after swallowing the medication and remaining in the upright position thereafter.

In conclusion, the present case has been an uncommon presentation of doxycycline-induced extensive ulcerations, mimicking esophageal cancer in two esophageal segments. A modification on the behavior of taking drugs can prevent these unpleasant complications.

## Competing interests

The authors declare that they have no competing interests.

## Authors' contributions

VT performed endoscopic examination, took pictures and biopsies from the lesions. HS performed histopathologic examinations of the biopsies and took pictures of the tissues. NB interned the patient in the Internal Medicine Clinic and performed biochemical and hematological studies. BE: followed up the patient and a contrubutor in writing the manuscript. GT performed the control endoscopy after the therapy and was a major contributor in writing the manuscript. FD interpreted the patient data regarding the oncological disease. All authors read and approved the final manuscript.

## Consent sction

Written informed consent was obtained from the patient for publication of this case report and accompanying images. A copy of the written consent is available for review by the Editor-in-Chief of this journal.
